# Volunteer Leadership in Older Adults’ Self‐Guided Learning Groups in Taiwan: Motivations and Management Processes Through an Ecological Lens

**DOI:** 10.1155/jare/5146512

**Published:** 2026-04-22

**Authors:** Shu-Chuan Wu, Li-Fen Jang

**Affiliations:** ^1^ Department of Child Development and Family Studies, Tzu Chi University, Hualien, Taiwan (ROC), tcu.edu.tw

**Keywords:** motivation, older adults, self-guided learning, volunteers

## Abstract

As global populations age, active aging and lifelong learning have become critical policy objectives. In Taiwan, the Ministry of Education (MOE) promotes Older Adults’ Self‐Guided Learning Groups (OASLGs); however, the sustainability of these groups relies heavily on volunteer leaders. This study investigates the motivations and management processes of “Excellent Leaders” in OASLGs using a qualitative approach. Drawing on Bronfenbrenner’s ecological systems theory, we analyzed semistructured interviews with Eight Excellent leaders who agreed to be interviewed. The findings reveal that leadership motivations, specifically altruism, self‐growth, and expectancy value, are deeply embedded within the microsystem of personal fulfillment and the Mesosystem of community interaction. Key management processes identified include “learning by doing,” resource mobilization, and the cultivation of peer‐based trust. This study extends the ecological understanding of senior volunteering by demonstrating how the Exosystem (policy‐structured training) functions not merely as a distant backdrop but as an essential scaffolding that actively sustains the Microsystem (individual leadership agency). Implications for policy include the necessity of continuous, structured training and the fostering of local support networks to sustain volunteer engagement in aging societies.

## 1. Background

Aging society is a global issue. Many developed and advanced countries, such as America, England, Japan, and Australia, are concerned about social participation from older adults. Successful aging, productive aging, and healthy aging are related issues. The U3A (university of the third age) movement in the United Kingdom involves local and online member‐led groups for older adults no longer in the workforce. The emphasis is on learning, staying active, and having fun in voluntary, low‐cost social groups [1]. Although U3A has yet to be developed in Taiwan, the self‐guided and self‐help learning model has been influential.

The learning atmosphere for older adults has gradually expanded in the country since Taiwan’s Ministry of Education (MOE) built Senior Citizens’ Learning Centers in local regions in 2008. The centers attracted numerous older adults (aged above 55 years) to participate and learn together. An investigation by Wu, Li, Lin, and Dong [2] on adult education, formal and informal, showed the participation of those aged 65 to 80 was up to 21.07% vs. 19.97% in 2017. However, in a few years, the participants remained the same, and fewer new learners joined, which became a serious concern. This phenomenon was similar to the claims by Glendenning [3] that the opportunities and channels for older adults’ learning failed to meet their learning needs. Consequently, the movement of self‐guided learning for adults may become a global trend.

In this era, an increasing number of older adults have obtained higher educational degrees and arranged their retired lives by their initiatives. Moreover, they are willing to pay for what they want and desire to do. After the development and popularity of senior citizens’ learning centers in Taiwan, many older adults experienced and enjoyed the fun of learning. It was just the right time to encourage more older adults to run their self‐guided learning groups. Older Adults’ Self‐Guided Learning Groups (OASLGs) in Taiwan were developed in 2012 by the MOE derived from the concepts of study circles in Sweden [4], U3A in the United Kingdom [5], and the American Association of Retired Persons (AARP) [6]. Training of the leaders of the OASLGs began in 2012, with implementation beginning in 2016. To ensure successful programs, the MOE operated a pilot trial for recruiting volunteers and provided more than 36 h of training and practical exercises between 2016 and 2018. The whole training program consisted of three stages: recruitment and interview, participation in the training courses, and group management and assessment. At the second stage, those who had passed the interview would have completed 57 h of training courses. The 57‐h training program covered diverse topics ranging from aging theories to practical group management. After finishing all the courses, the candidates must submit their own self‐guided group management plan and explain how to execute their plans in their community. Every plan and execution required a total of 24 h of practical group leading with an option of eight three‐hour sessions or twelve two‐hour sessions. During the implementation, advisors would support the candidates during and after the program. After the completion of all requirements and assessments, each participant would receive the certificate of qualification issued by the MOE. The efforts encouraged volunteers to organize and run OASLGs in their communities. There was a particular focus on recruiting older adults (aged above 55 years) living in suburban areas that lacked learning resources. This informal, flexible, and localized learning style was created to attract more older adults to join. By 2022, 642 leaders have been trained [7]. Consequently, more older adults have enjoyed local opportunities for learning and social participation.

Qualified leaders can apply and receive subsidies from the MOE to operate learning groups. The leaders must design their plans first, recruit the older adults in their communities, and manage the learning activities with group members. Now, many Excellent Leaders run self‐guided groups every year. However, matching the members’ learning needs, caring for them well, and maintaining participation are not easy tasks.

Despite the growth of OASLGs, existing literature has predominantly focused on the benefits for learners rather than the experiences of the leaders who sustain these groups. Leading a peer group involves complex challenges, from curriculum design to conflict resolution. Little is known about the deeper motivations that drive these volunteers to persist, nor how they navigate the management process within their specific social contexts. This study addresses this gap by examining the experiences of “Excellent Leaders” through the lens of ecological systems theory, identifying the multilevel factors that contribute to sustainable volunteer leadership.

Leading OASLGs requires significant dedication to a complex management process, which encompasses participation in rigorous training programs, the design of learning plans, member recruitment, and ongoing group operations. Consequently, understanding the drivers behind this commitment is essential. That is why we desired to explore this study. Moreover, the Excellent Leaders’ stories may offer new leaders valuable guidelines and the impetus for continued support by the MOE. The purposes of this study are as mentioned above.

## 2. Literature Review

Self‐directed or self‐guided learning is the central concept in this study. The literature review focuses on four parts: self‐guided learning in older adults, OASLG in Taiwan and related studies, volunteering and motivations to volunteers, and the ecological system theory.

### 2.1. Self‐Guided Learning in Older Adults

The concepts of self‐directed learning, self‐guided learning, informal learning, or heutagogy are very similar. Self‐directed learning, proposed by M. S. Knowles in 1975, is not new and has created confusion as many related concepts are often used interchangeably or in similar ways. Some examples of similar concepts include self‐directed learning, self‐planned learning, learning projects, self‐education, self‐teaching, autonomous learning, independent study, and open learning [8]. Self‐directed learning has been defined as a process of constructing personality and an environmentally determined phenomenon. It is the process in which individuals take the initiative, either with or without the help of others, in diagnosing their learning needs, formulating the learning goals, identifying human and material resources for learning, choosing and implementing appropriate learning strategies, and evaluating learning outcomes [9, 10]. This process involves three aspects of interaction. The first aspect is the application of some actions or procedures; the second is by a person who is not psychologically averse to the experience; and the last is in an environment which, at the very least, does not preclude the emergence of self‐directed learning [9, 10]. However, Bengry [11] stressed the importance of collaboration in self‐guided learning and the revolution of self‐directed learning in the twenty‐first century. Self‐guided learning is more flexible in learning plans, learning styles, and learning environments and focuses on peer learning.

### 2.2. OASLG in Taiwan and Related Studies

In light of flexibility, the MOE in Taiwan has decided to use the concept of a self‐guided learning group in which leaders are essential. Older adults must maintain a warm learning environment [12], guiding members to share and learn [13, 14].

The primary purpose of building OASLGs in Taiwan is to encourage them to develop and be in charge of their learning opportunities, learning interests, and learning styles and to encourage them to perform their autonomous and self‐help learning abilities. This learning model includes self‐help, self‐directed, and self‐guided learning concepts, named “Older Adults’ Self‐Guided Learning Group” by the MOE.

Older adults joining the self‐guided learning groups are encouraged to determine their learning plans. The program is a student‐centered learning method emphasizing peer‐to‐peer learning experiences where group leaders encourage group members to share their experiences, knowledge, and interests.

The implementation of OASLGs in Taiwan was examined by Huang et al. [15]. The researchers reported that most group leaders wanted to benefit others and themselves and that the self‐guided learning groups were meaningful and deserved to develop sustainably. The leaders successfully recruited 1144 older adults. The first‐time participants represented 73% of adults attending the senior centers. The ratio of the first‐timer increased to 76% in 2019. The results indicated that older adults in Taiwan are interested in this flexible learning style, similar to the findings in Poland by Gierszewski and Kluzowicz [16] and in Australia by Hebestreit [17].

Dixson [18] claimed that learning is not limited to obtaining accurate information or answers from others but finding the true meaning from experiences. Nowadays, aging populations are increasing worldwide; it is also the time for older adults who are retired from work to develop their own interests and cultivate self‐guided learning [19]. The OASLGs in Taiwan have been constructed based on the concept that older adults get control of their own learning goals, learn what they need, and can organize it through self‐help and self‐guided management [13, 14, 20].

The OASLG leaders have recruited 10 to 15 older adults to share their learning interests, create learning styles and opportunities, and establish their learning groups. In the process, older adults engage in social activities, build self‐esteem and self‐confidence, and develop better and more extensive relationships. Yang [14] claimed that OASLGs in Taiwan consist of seven characteristics: automatic, meeting older adults’ psychological development, free from space and time limits, more flexible, economic traits, benefiting others, individualized, and accessible expansion. The related research by Lin [21] on OASLG revealed the following: Leaders were primarily female (83.9%), aged 50–69 years (77.5%), and retired (52.8%). A study by Huang [12] suggested that leaders must keep learning and empowering themselves and that group leaders can make life more abundant and meaningful. By leading the learning groups, both leaders and group members experienced achievements and learning [12, 13, 20].

Unlike the Western U3A model, which relies heavily on bottom‐up member autonomy and independence from government intervention [5], the Taiwan OASLG model operates within a unique “government‐supported” framework. This distinction is critical; while Western studies emphasize individual agency, the sustainability of Taiwan OASLG depends on a specific “top‐down empowerment” strategy where the government (Exosystem) actively scaffolds local leadership. Therefore, applying Western concepts of self‐directed learning requires adaptation to account for this intense policy‐driven context.

### 2.3. Volunteering and Motivations to Volunteer

There are many opportunities and types of volunteer services in Taiwan. Regarding the motivation to participate in service, Zeng and Zeng [22] indicated that the two factors of volunteer participation motivations are social orientation and personal growth orientation. According to the research on volunteers’ participation in Taiwan, the motivations of volunteers are diverse. However, Lee et al. [23] listed five motivations of value recognition, social service, self‐growth, achievement, and being influenced by others. Many volunteers have expressed that their lives have become more prosperous through the volunteering experience, where they participated in more social interactions, had the opportunity to work with different age groups, and participated in various activities. In conclusion, volunteer participation is not only beneficial to personal learning and growth but also confirms self‐worth and affirmation through personal interaction and altruism. As a result, the participation rate of volunteers in Taiwan is high.

While existing studies [22, 23] effectively identify why individuals initiate volunteering, such as altruism and self‐growth, they offer limited insight into how leaders sustain their commitment amidst the complex management challenges of OASLGs. Motivation theory tends to view the individual in isolation. However, considering the policy involvement in Taiwan, explaining leadership sustainability solely through individual psychological traits is insufficient.

### 2.4. The Ecological Systems Theory

To bridge the gap between individual motivation and systemic support identified above, this study adopts Bronfenbrenner’s framework not merely as a descriptive tool, but as an analytical lens. Unlike linear motivation theories, the ecological perspective allows for a synthesis of distinct factors, enabling us to examine how the Exosystem (MOE policy/training) and Mesosystem (family support) actively interact with the Microsystem (individual leadership). This approach transforms the analysis from a list of motivations to a dynamic map of interactions that sustain volunteer energy.

The ecological systems theory, a significant theoretical perspective, places human development in an environmental context. It views an individual’s experiences as nested within an interconnected system [24]. The socioecological model, introduced in the 1970s and formalized as theory in the 1980s, provides a valuable framework for understanding human development. Applying this ecological approach to the development of older adults in a self‐guided learning group, we can gain insights into the complex inter‐relationships that shape their learning experiences.

Ecological systems theory places the development/learning of older adults in an ecological perspective, in which an individual’s experiences are nested within an interconnected system. The ecological approach emphasizes the interconnection of the influence on personal development. Bronfenbrenner [25] states that social, political, biological, and economic circumstances also affect older adults.

**FIGURE 1:**
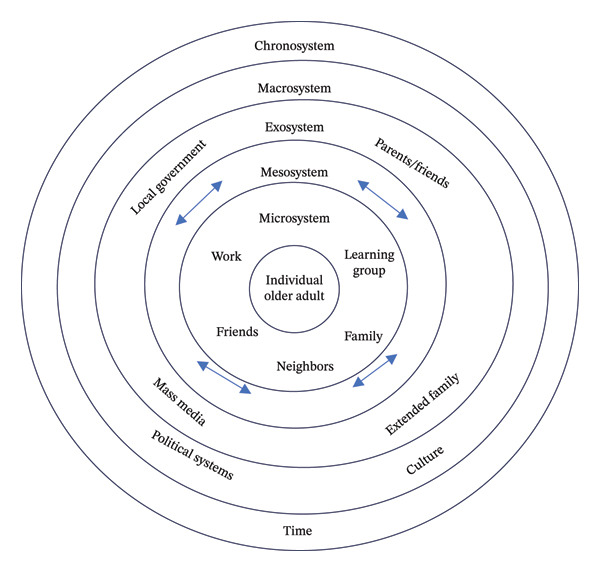
The diagram of Bronfenbrenner’s ecological systems which was revised from the version developed by Guy‐Evans [26].

The conceptual framework of the ecological perspective is composed of four levels, and it has been modified into five levels: Microsystem, Mesosystem, Exosystem, Macrosystem, and Chronosystem [26]. The revised diagram of Bronfenbrenner’s ecological systems theory is listed as below (Figure 1). These levels are not isolated, but rather, they are mutually influential. Each level is assumed to be related, and changing one part of the system can significantly impact other parts. This intricate interplay underscores the complexity and depth of the ecological systems theory, inviting researchers and educators to delve deeper into this rich and multifaceted topic.

Microsystems refer to the complex relations between the developing person and the environment in the immediate setting containing that person [27]. For example, OASLGs are characterized by face‐to‐face connections among individuals who may be friends, family members, learning partners, or community neighbors. The microsystem refers to the direct interactions with a social agent, which includes personal growth, learning courses, and training opportunities. Mesosystems include the interrelations of primary settings connecting the developing person at a point in his or her life. For older adults, it may include different backgrounds, such as educational, cultural, and economic backgrounds. For instance, education may impact how people select learning programs and training opportunities. The levels of education may influence individuals’ motivations and capabilities to contribute to volunteer service due to variations in their knowledge, abilities, and socioeconomic status. In addition, personal background, such as financial and cultural, may influence the opportunities for social participation and their learning motivations.

According to Bronfenbrenner [25], such environments external to the developing persons are ecosystems. An Exosystem contains formal and informal social structures [27]. The Exosystem may indirectly affect older adults, such as policies of recruiting OASLGs. There are other domains indirectly related to their opportunities, such as opportunities and training resources that are supportive of the policies from MOE.

When interpreted from an ecological perspective, these mesosystems and ecosystems, such as personal background and self‐guided learning groups in which the policy may not directly influence or participate, still influence the lives of older adults. The fourth ecological layer is the Macrosystem, which includes ideologies and attitudes of the culture, such as laws, morals, values, customs, and world views [25, 28]. Although Macrosystems are not readily part of the immediate world of older adults, they are impacted by them. For example, the values, expectations, motivation, self‐achievement, and attitudes of altruism directly affect the individuals’ well‐being. Therefore, the opportunities for social participation and social support, such as learning groups, play essential roles for both the individual and the society. The outermost layer is the chronosystem, which focuses on the role of time and how it impacts older adults’ development, including life transitions and societal changes [28]. Excellent leaders of OASLGs not only have great enthusiasm, but also accumulate rich experience through long‐term commitment and accompany senior adults. They learn together and enrich each other’s life journey.

This study utilizes Bronfenbrenner’s ecological systems theory as an analytical lens to understand the dynamic interaction between the leaders and their environments. This framework guides our data analysis by allowing us to map how personal motivations are sustained or constrained by external resources and social values over time.

## 3. Method

OASLG is a new attempt to prompt older adults’ opportunities. This study explores the motivations for organizing learning groups and the management process of excellent leaders and discovers what they experienced and learned through the process. This is an exploratory research. The current study adopted a qualitative approach [29]. One‐to‐one interviews were used to explore the leaders’ motivations for planning, organizing, and leading the learning groups as well as their management processes. To ensure the quality, breadth, and richness of the collected information and conclusion, data from the one‐on‐one interviews and the plans, diaries, and reflections from group leaders and various other sources are analyzed.

### 3.1. Participants

The participants of this study were Excellent leaders selected by the MOE. According to the data offered by the MOE, there were 491 qualified leaders. Those designated as Excellent included 45 selected in 2017, 40 in 2018, and 21 in 2019. Those who received the Excellent Accreditation for at least two years were recruited for interviews. Less than 20 leaders met this condition. Two excellent leaders from each of the four districts in the North, Central, South, and East were interviewed in Taiwan to collect urban and rural data. The sample size of eight was determined by data saturation, where no new themes emerged from subsequent interviews. Given that strictly fewer than 20 leaders met the “Excellent” accreditation criteria for at least two years, this sample represents a significant portion of the specific target population.

Six of the eight interviewees had previous experience in education, such as kindergarten, after‐school programs, or elementary school. The majority have been volunteers or have accompanied older adults. All eight participants had obtained at least an undergraduate college degree. Half of the interviewees had prior program planning experiences, but half reported that it was their first‐time building and implementing a group plan. The ages of the participants ranged from 40 to 70 years.

### 3.2. Instrument

Because of the new try to explore the motivations and the process of OASLG leaders running the groups, this study adopted semistructured interviews. Elite interviews were conducted [30]. The interview questions were examined by a scholar and a practical expert to ensure clarity and ease of administration. The final interview guide consisted of three parts. Part one included demographic background, such as gender, age, educational background, and work experience. The second part focused on the experiences with OASLGs, including their motivation, how they organized and led the groups, their leadership processes, and lastly, reflection and feedback. The complete interview outline is detailed in the Appendix. Ethical standards were strictly adhered to throughout the research process. This study was aligned with institutional ethical guidelines regarding nonmedical human subject research. Written informed consent was obtained from all participants before the interviews, ensuring their anonymity, confidentiality, and right to withdraw from the study at any time.

### 3.3. Research Process and Data Collection

Data were collected using semistructured interviews. The sample size was determined by the principle of data saturation, defined as the point where no new themes or codes emerged from subsequent interviews [31]. After analyzing the eighth interview, the researchers agreed that thematic saturation had been reached. Given that fewer than 20 leaders in Taiwan met the stringent “Excellent” accreditation criteria for at least two years, this sample of eight represents a significant and representative portion (approximately 40%–50%) of the specific target population.

Each interview lasted 120–150 min, allowing for deep engagement. During the interviews, questions followed the designed outline, but free, flexible, and exhaustive answers were encouraged to allow participants to share their lived experiences fully [32]. The entire process was audio‐recorded with permission. To ensure data accuracy (member checking), interviewees received the transcripts to verify reliability and provided additional documents, such as reflections on group learning.

### 3.4. Data Analysis

To ensure the trustworthiness and credibility of the analysis, investigator triangulation was adopted [33]. Two researchers with backgrounds in adult education and family studies independently coded the transcripts to minimize personal bias. Following Kao’s [34] “whole‐part‐whole” approach, we first read the transcripts holistically to grasp the overall context, then conducted line‐by‐line open coding to identify initial concepts, and finally grouped these codes into themes. Discrepancies in coding were resolved through regular peer‐debriefing meetings until a consensus was reached.

The analysis also utilized the constant comparative method [31]. The researchers followed three steps: (1) immersing in the data to understand the context; (2) applying the hermeneutical circle [34, 35] to organize individual contexts into themes; and (3) interpreting the phenomena to reflect the participants’ actual experiences accurately. The rigorous process minimized personal bias and ensured that the interpretation accurately reflected the participants’ experiences.

### 3.5. Ethical Considerations

A full disclosure regarding formal Institutional Review Board (IRB) approval is necessary. At the time of this study’s initiation, formal IRB approval was not strictly mandated for nonclinical, minimal‐risk social science research involving adult volunteers in Taiwan. Consequently, formal IRB approval was not obtained before data collection.

However, the research process strictly adhered to standard ethical guidelines for human subject research. All participants were competent adults (aged 40–70 years) who voluntarily managed OASLGs. The semistructured interviews posed minimal to no psychological or physical risk to the participants. Before the interviews, written informed consent was obtained from all individuals. Participants were fully informed about the study’s purpose, their right to withdraw at any time without consequence, and the measures taken to ensure the confidentiality and anonymity of their data. Furthermore, to protect their rights and ensure accuracy, participants were provided with the interview transcripts for review and verification before data analysis.

## 4. Data Availability

To ensure the confidentiality and privacy of the participants, all digital files—including audio‐recordings and de‐identified transcripts—are securely stored on password‐protected computers accessible solely to the research team. In accordance with institutional research ethics guidelines, the data will be retained for 5 years. Given the qualitative nature of the semistructured interviews and the sensitivity of the participants’ personal experiences, full transcripts are not publicly available. However, anonymized data supporting the findings of this study are available from the corresponding author upon reasonable request.

### 4.1. Limitations

Less than 20 leaders met this condition. To collect both urban and rural data, two Excellent leaders from each of the four districts in the North, Central, South, and East were interviewed in Taiwan. Since the sampling required the consent of the interviewees, the selection of gender, varied educational levels, and ages were not able to be well balanced, which has become one of the limitations of this study.

Second, it is an exploratory research design, and semistructured interviews are adopted. The results are not sufficient to draw inferences.

## 5. Results

This study aimed to explore why the leaders of OASLG led their groups in Taiwan and their leadership process. This study adopted semistructured interviews; 8 excellent participants shared their motivation to engage in program planning and implementing their OASLG and their leadership experience.

### 5.1. Motivations

Motivations of the leaders engaging in program planning and implementing the OASLG included altruistic motivation, growth motivation, and expectancy‐value motivation.

#### 5.1.1. Altruistic Motivation

Participants claimed they hoped they could accompany and offer some support and service for older adults in the community. That way, they were energized by offering learning activities and felt happy and accomplished while volunteering. Older adults’ smiles and encouragement gave the leaders the will and power to continue their group management and to offer more services.

*I felt happy I could really help them (M02).*

*I hope they infect with a happy atmosphere when we learn together (E01).*

*I asked them to participate in, and learn some knowledge (S02).*

#### 5.1.2. Growth Motivation

Participants reflected on their shortcomings and weaknesses and tried to learn more and do more for older adults.

*I must keep learning, and then I can get more ideas on arranging the activities (N02).*

*I needed to improve, so I tried to learn in various fields (M02).*

#### 5.1.3. Expectancy‐Value Motivation

Participants mentioned that they eagerly engaged in the training and led the learning groups to enhance their abilities and experiences, develop their talents, and build their value.

*I felt terrific and surprised that I could manage a group and complete so much business. That is really unexpected (E01).*

*Serving the older adults now means offering the service to ourselves many years later. I started to anticipate my future self (S01).*

The main motivation of the participants who engaged in the program planning and held the learning groups was altruistic motivation. When they felt and saw the needs of older adults in the community, they hoped and wanted to do something for them, share, and offer more services. The second and third participation motivations were self‐growth and expectancy value. The participants expected that they could enhance their knowledge, abilities, and services to meet the older adults’ learning needs. So, during the process of group learning, they found their accomplishment and value. When the members’ approval and trust stimulated them, they would highly rely on learning and acquire more energy leading the groups. The result of this study responded to the ecological theory [24] that every individual’s experience was nested within an environmental system; in a microsystem, older adults could achieve happiness and self‐growth when they met, shared experiences, and learned together in their learning groups. In the Mesosystem, the leaders of OASLGs with higher socioeconomic status were willing to serve the opportunities for older adults through altruistic motivation, self‐growth, and expectancy‐value motivation, especially in suburban areas.

### 5.2. Leadership Processes

How do you lead the learning group successfully, from new learners to excellent ones? According to the analysis of interviews, this study concluded that there were six critical points for successfully leading a group.

#### 5.2.1. Learning by Doing

The first one, leaders valued learning by doing and focused on gradually exploring older adults’ talents and potential. “*Thanks for all members’ participation, I learned a lot. To offer older adults more successful and happy learning, I paid more attention to learning (M02); This is my first time finishing a project by myself, understanding the related knowledge about older adults, running the whole group, and then arriving here (E02); I joined another group and learned how to manage (S01).*” Although the participants might have volunteered experiences, leading a learning group was their first time. It was crucial for them to learn by doing, serving, and being with older adults.

#### 5.2.2. Multidimensional Promotion and Recruitment

The second one, leaders paid attention to promotion and recruited older adults in communities. “*I designed the DM myself, asked the Village Chief to help me, and marketed it on social media (N01-08); I recruited them door to door (S02); I asked my sisters, neighbors, and friends to join (E01).*” Leaders used various methods to recruit older adults to form self‐guided learning groups in their community.

#### 5.2.3. Family Support and Member Participation

The third one, older adults’ participation and family support, was the biggest motivation for running groups. “*Older adults shared their learning experiences with their friends…they were enthusiastic in participating (M02); I thanked my husband, my most important support (N01). Their happy smile encouraged me. It was amazing (E02). My sisters were all retired, and they supported me and helped me with marketing my learning group (E01)*.” When leaders wanted to build up their learning group, they would recruit their retired family, neighbors, and community members. Family support offered leaders positive stimulation and encouragement.

#### 5.2.4. Connecting Resources and Building Trust Relationship

The fourth one, leaders connected with local resources and built trusting relationships. “*They said, “OK! You just do it! We all support you,” which impressed me and encouraged me (N01); I got a lot of support from group members (M01); The community chief helped me a lot. He recruited lots of members. I was happy I can help more older adults towards successful ageing even though I have retired (M01); I expected the learning activities here every week to be fun and exciting (E01); Finally, my mother-in-law joined as our members and was enthusiastic (M02).*” The nice and warm atmosphere and relationship of group learning together, mutual support, and encouragement were the greatest feedback for leaders.

#### 5.2.5. The Virtuous Cycle of Service and Achievement

The fifth one, leaders worked hard to benefit others and then accomplished more. “*I started to serve others as a volunteer, which became our consensus (N02); at first, I just offered learning services for older adults, and now I have more and more opportunities (E02); the most important thing for leaders is to make efforts to serve other people willingly. That is what I thought (E02); we did everything happily without any rewards (S02).*” It proved that volunteering brought happiness for everyone.

#### 5.2.6. Peer Learning and Sharing

The sixth one, leaders would explore older adults’ talents and let them perform and share the spirit of peer learning with others. “*My male group members were charming and sincere because they really enjoyed learning with us, gave the group extensive support, and sometimes gave us advice (N01); our team members were all teachers. We shared. What I did was only to plan the program and act it out (N02); I would ask members to demonstrate or share what they understood or mastered, which made them feel a sense of achievement (M01).*” It matched Bengry’s [11] concept that learning groups for older adults stressed the importance of collaboration and self‐guided learning. The leaders of OASLG would try to understand more about older adults and better interact with them. “*I let them know we all had to keep learning, and I tried to help them figure out they were still valuable. (E02); Older adults would keep learning together when they felt valued and respected (M02).*”

To sum up, becoming an Excellent Leader is a journey of growth. It begins with learning by doing and finding members through active promotion. With family support, leaders move forward to connect resources and build trust in the community. They soon realize that serving others brings personal achievement—a virtuous cycle. Ultimately, this process creates a space for peer learning, where every older adult feels valued and empowered to share.

## 6. Discussion

The participants were between 40 and 70 years of age. They were college‐educated and enjoyed being volunteers. This finding was similar to Hebestreit’s [17] research on U3A in Australia. Volunteers aged 40–70 years with higher educational degrees would pay attention to serving older adults.

This study concluded that the participants had three motivations: altruism, self‐growth, and expectancy value. The main motivation of participants involved in the program planning and conducting the learning groups was altruism. When the participants felt and saw the needs of the older adults in the community, they desired to do something for them, share, and offer more services for them. The volunteers could reexamine their life experiences, accumulate their energies, and ensure their life value, especially after they offered services or benefits to others with selfless dedication [36–38]. The second and third participation motivations were self‐growth and expectancy value. The participants expected that they could enhance their knowledge, abilities, and services to meet the learning needs of older adults. So, during the process of group learning, they found self‐accomplishment and value. When the members’ approval and trust stimulated them, they would engage highly in learning and leading the groups. The leaders had to keep learning, empowering, and dedicating themselves [12, 23].

This study revealed that family support and the amiable group learning atmosphere would encourage the leaders to keep participating. This finding was similar to the results of the relevant research on older adult volunteers by Fisher and Schaffer [39], Hickson and Housley [40], Lo [41], and Tsai [42]. Moreover, Ma [43] suggested that older adults’ learning was prompted by their family support and encouragement, which was the same as this study. The findings of the current study indicated that the leaders performed self‐directed learning and integrated their past experiences and resources in organizing their self‐guided learning groups, which met the concepts and operational spirit of OASLG promoted by the MOE in Taiwan.

Although all the leaders adopted different integrating skills and resources for marketing, recruiting members, and developing their learning activities, they all brought learning opportunities and resources into communities successfully and sincerely. Moreover, they all benefited others and received advantages from it simultaneously. This study also found that most leaders directed most of the team’s operations, structure, and responsibilities but maximized peer learning and team discussions when the groups became more stable. These processes met the goals and functions of building self‐guided learning groups [14, 44].

The participants announced their roles and tasks and developed self‐guided and peer learning for community older adults. This research verified that the leaders who were interviewed adhered to the spirit of sharing, respect, encouragement, and mutual stimulation as listed in the guidelines of the leader’s handbook edited by the MOE [45].

According to the participants’ interviews, the following recommendations could help the group leaders organize, manage, and run the group more successfully. The recommendations included the following: (1) Leaders must be full of passion to benefit others and will to work for them; (2) leaders should understand older adults more and spend time being present with them; (3) leaders must recruit and encourage community older adults to participate in learning group; (4) leaders should explore and emphasize on older adults’ talents and potential; (5) leaders must connect with local resources and build trusting relationship; and (6) leaders should let older adults perform and share with the spirit of peer learning.

These recommendations can be divided into a leader’s personality and practical activities. The leader’s personality, spirit of altruism, passion, love, and the will to know and be with the older adults were really valued. Regarding practical activities, leaders must keep lifelong learning, learn by doing, integrate community resources and trust, and develop peer learning. In some suburban areas, those older adults with low educational levels or socioeconomic backgrounds had low self‐directed learning ability or motivation. Group leaders tended to guide them to learn first and gradually guide them to demonstrate their expertise and abilities. During the long‐term management of the learning groups, leaders often established good relationships and obtained resources in the community. Leaders could develop more trust and support and recruit more members. The result of this study responded to the ecological theory that every individual’s experience was nested within an environmental system; in a microsystem, older adults could achieve happiness and self‐growth when they met, shared experiences, and learned together in their learning groups. In the Mesosystem, the leaders of OASLGs with higher socioeconomic status were willing to serve the opportunities for older adults through altruistic motivation, self‐growth, and expectancy‐value motivation, especially in suburban areas. Exosystem, OASLGs in Taiwan, was launched and supported by MOE. The free training courses and application system allowed the leaders to learn, organize, and act out the service. At last, Macrosystem, by participating in the OASLGs, the leaders and older adult members could reveal their own and older adults’ talents and potential, build trusting relationships, enjoy peer‐learning experiences, and connect with more community resources. The results of the study approved the findings and applied the ecological theory.

Using ecological systems theory as an analytical lens, we conceptualize the volunteer experience across four levels: the Microsystem, involving the leader’s intrinsic motivations and immediate interactions with group members; the Mesosystem, encompassing the interrelations between the leader’s family support and community networks; the Exosystem, representing the MOE’s policy structures and training resources which indirectly but powerfully shape leadership capacity; and the Macrosystem, reflecting broader cultural values regarding aging and volunteering.

This study extends Bronfenbrenner’s framework by situating it within the unique context of the Taiwan OASLG initiative. Our findings reveal a distinct mechanism of “Policy‐Driven Agency.” In this context, the Exosystem—specifically the MOE’s structured training and funding—does not merely influence older adults from a distance; rather, it functions as essential “institutional scaffolding” that actively constructs the Microsystem—the leaders’ capacity to manage autonomous groups.

This finding refines the theoretical understanding of Excellent Leaders by demonstrating that in government‐supported aging societies, individual motivations, such as altruism, self‐growth, and expectancy value, are insufficient on their own. They require a direct, empowering connection with the Exosystem to be sustained. Thus, successful volunteer leadership in OASLGs is not just an expression of personal agency, but a symbiotic outcome of individual drive supported by top‐down governmental empowerment.

## 7. Conclusion

This study investigated the motivations and management processes of Excellent Leaders in Taiwan’s OASLGs. Based on the qualitative evidence, we conclude that when the leaders initially accompanied their family members or engaged in learning activities, they discovered older adults’ needs and witnessed the “healing power” of being together. Consequently, they volunteered to participate in the OASLG training programs provided by the MOE. Moreover, they all affirmed the effectiveness of OASLG’s training programs. They acquired multiple advantages from program training, integrating various resources, and recruiting and managing learning groups. This study confirms that the first and foremost motive for leading the groups was altruism, followed by self‐growth and expectancy value.

Regarding the management process, evidence indicates that successful leaders possess high educational backgrounds and utilize a “learning by doing” approach. They focused on exploring suitable management styles, connecting with community resources, and developing peer learning. By leading the learning groups, the participants were trusted by the group members, benefited by serving older adults, and built their confidence and group management experiences. Concerning the management process, the leaders had to maintain lifelong learning, learning by doing, exploring suitable management for community older adults, connecting with community resources, and developing peer learning with members. Critically, the ecological analysis reveals that leadership sustainability is not solely an individual trait but a result of supportive interactions between family (Mesosystem), community resources (Exosystem), and policy support (Macrosystem).

## 8. Recommendations for Policy and Practice

Based on the findings above, we offer the following forward‐looking suggestions for policy‐makers and practitioners: (1) Strengthen Institutional Scaffolding (Exosystem): The MOE should continue and expand specific training programs, as the data show these are not just educational but essential for empowering leaders to handle management challenges. (2) Foster Local Support Networks (Mesosystem): Future programs should explicitly encourage leaders to recruit “support teams” involving family members or neighbors, rather than working in isolation, to prevent burnout. (3) Promote Peer‐Learning Models: Guidelines for new leaders should emphasize the “learning by doing” and “sharing” approach verified in this study, transforming the leader’s role from a teacher to a facilitator.

## Funding

No funding was received for this manuscript.

## Conflicts of Interest

The authors declare no conflicts of interest.

## Data Availability

The data that support the findings of this study are available from the corresponding author upon reasonable request.
